# Chromosomal Aberrations in Pediatric Patients with Developmental Delay/Intellectual Disability: A Single-Center Clinical Investigation

**DOI:** 10.1155/2019/9352581

**Published:** 2019-11-06

**Authors:** Ting Hu, Zhu Zhang, Jiamin Wang, Qinqin Li, Hongmei Zhu, Yi Lai, He Wang, Shanling Liu

**Affiliations:** ^1^Department of Obstetrics & Gynecology, West China Second University Hospital, Sichuan University, Chengdu, China; ^2^Key Laboratory of Birth Defects and Related Diseases of Women and Children (Sichuan University), Ministry of Education, Chengdu, China

## Abstract

**Introduction:**

Chromosomal microarray analysis (CMA) has currently been considered as the first-tier genetic test for patients with developmental delay/intellectual disability (DD/ID) in many countries. In this study, we performed an extensive assessment of the value of CMA for the diagnosis of children with ID/DD in China.

**Methods:**

A total of 633 patients diagnosed with DD/ID in West China Second University Hospital, Sichuan University, were recruited from January 2014 to March 2019. The patients were classified into 4 subgroups: isolated DD/ID, DD/ID with multiple congenital anomalies (MCA), isolated autism spectrum disorders (ASDs), and DD/ID with epilepsy. CMA was performed on Affymetrix 750K platform.

**Results:**

Among the 633 patients, 127 cases were identified as having pathogenic copy number variations (pCNVs) with an overall positive rate of 20.06%. Of the 127 cases with abnormal results, 76 cases had 35 types of microdeletion/microduplication syndromes (59.84%) including 5 cases caused by uniparental disomy (UPD), and 18 cases had unbalanced rearrangements (14.17%) including 10 cases inherited from parental balanced translocations or pericentric inversions. The diagnostic yields of pCNVs for the subgroups of isolated DD/ID, DD/ID with MCA, isolated ASD, and DD/ID with epilepsy were 18.07% (60/332), 34.90% (52/149), 3.70% (3/81), and 16.90% (12/71), respectively. The diagnostic yield of pCNVs in DD/ID patients with MCA was significantly higher than that of the other three subgroups, and the diagnostic yield of pCNVs in isolated ASD patients was significantly lower than that of the other three subgroups (*p* < 0.05).

**Conclusion:**

Microdeletion/microduplication syndromes and unbalanced rearrangements are probably the main genetic etiological factors for DD/ID. DD/ID patients with MCA have a higher rate of chromosomal aberrations. Parents of DD/ID children with submicroscopic unbalance rearrangements are more likely to have chromosome balanced translocations or pericentric inversions, which might have been missed by karyotyping. CMA can significantly improve the diagnostic rate for patients with DD/ID, which is of great value for medical management and clinical guidance for genetic counseling.

## 1. Introduction

Developmental delay/intellectual disability (DD/ID) affects approximately 3% of the general population [1]. In China, 11,820,000 people were diagnosed with DD/ID, of whom 954,000 were younger than 6 years of age [2]. Taking care of a patient with DD/ID exerts a substantial financial and emotional burden on his/her family and society. Approximately more than half of DD/ID cases resulted from genetic etiologies, including chromosomal abnormalities, microduplication or microdeletion syndromes, and monogenic disorders [3]. Other etiologies include teratogenic exposures, perinatal asphyxia, infections, etc. [4].

Submicroscopic chromosomal aberrations (copy number variants, CNVs) play a significant role in the pathogenesis of DD/ID, and the diagnostic yield of chromosomal microarray analysis- (CMA-) detected CNVs associated with these disorders ranges from 12% to 29% [5–8]. Currently, the clinical utility of CMA has been recognized by several professional societies and has been recommended as the first-tier genetic test for patients with unexplained DD/ID, autism spectrum disorders (ASDs), and/or multiple congenital anomalies (MCAs) [9–12]. In this study, we investigated 633 Chinese children with unexplained DD/ID combined with other conditions by the Affymetrix® CytoScan™ 750K Array over a period of 5 years and extensively assessed the value of CMA for the diagnosis of children with DD/ID.

## 2. Methods

### 2.1. Patients

A total of 633 Chinese patients with varying degrees of DD/ID (359 males; 274 females), with ages from 3 months to 17 years, were recruited from the Department of Neurological Rehabilitation at West China Second University Hospital, Sichuan University, from January 2014 to March 2019. All patients were classified into 4 subgroups: isolated DD/ID (*n* = 332), DD/ID with MCA (*n* = 149), isolated ASD (*n* = 81), and DD/ID with epilepsy (*n* = 71).

The detailed evaluations of the patients included prenatal/birth history, family history, pedigree, physical examinations, and imageological examination. The inclusive criteria were as follows: DD/ID diagnosed according to the Diagnostic and Statistical Manual of Mental Disorders, Fifth Edition (DSM-5) with IQ/DQ < 70 assessed by the Gesell Development Scale, the Wechsler Preschool and Primary Scale Intelligence, or the Wechsler Intelligence Scale for Children. The exclusion criteria were as follows: (1) history of hypoxia, toxication, central nervous system infection, and cranial trauma; (2) evidence of recognizable inherited metabolic disorder; (3) typical clinical manifestation of Rett syndrome for female patients; (4) mutations in the FMR1 gene for male patients; and (5) fetus or newborns with multiple malformations.

The peripheral blood samples of the patients were analyzed by CMA. Informed consent was obtained from their mentally healthy parents before detection. In addition, the peripheral blood samples of their parents underwent CMA to determine whether the CNVs of the patients were inherited or *de novo* to determine the clinical significance. The research was approved by the Medical Ethics Committee of West China Second University Hospital, Sichuan University.

### 2.2. Chromosomal Microarray Analysis

Whole genomic DNA was extracted from peripheral blood cells of each patient and his or her parents using QIAamp DNA Blood Mini Kit (Qiagen, Valencia, CA, USA) and subjected to CMA-single nucleotide polymorphism (SNP) array analysis by using the Affymetrix® CytoScan™ 750K Array (Affymetrix, Santa Clara, CA, USA). The procedure was described in our previous publication [13].

When the fragment size of absence of heterozygosity (AOH) was larger than one-third of the chromosome, analysis software UPD tool_0.2 was used to separate the AOH into uniparental disomy (UPD) or consanguinity by comparison with the parental results.

The detected CNVs were systematically evaluated for clinical significance. The procedure was also described in our previous publication [13].

### 2.3. Chromosomal Karyotyping

When a gain and a loss of more than 5 Mb were simultaneously detected at one end of two different chromosomes or at the both ends of a single chromosome in one sample, peripheral blood samples of the normal parents were karyotyped to confirm whether the parents had chromosomal balanced translocations or inversions.

### 2.4. Statistical Analysis

Statistical analysis was performed by using SPSS software, version 24. The frequency of pCNVs was compared among subgroups of isolated DD/ID, DD/ID with MCA, DD/ID with ASD, and DD/ID with ASD by using the chi-square test. A value of *p* < 0.05 was considered to indicate statistical significance.

## 3. Results

### 3.1. Diagnostic Yields of pCNVs

We detected 149 pCNVs (including 5 UPDs) in 127 cases (65 males; 62 females), accounting for 20.06% of the series (Table 1). These pCNVs, including 100 deletions and 44 duplications, were highly variable in size, ranging from 223 kb to 102,400 kb (Table 2).

Fifty-two pCNVs (34.90%, 52/149) were detected in patients with MCA. In the subgroup of MCA, several clinical manifestations were found, including facial dysmorphic features, growth disorders, micro/macrocephaly, cleft palate, ear deformity, abnormal hands or feet, abnormal heart morphology, and abnormal genital system. In addition, 60 pCNVs (18.07%, 60/332) were detected in patients with isolated DD/ID, 3 pCNVs (3.70%, 3/81) were detected in patients with isolated ASD, and 12 pCNVs (16.90%, 12/71) were detected in patients with epilepsy. The proportion of pCNVs detected in patients with MCA was significantly higher than that in patients with isolated DD/ID (*p* ≤ 0.001 (34.90% vs. 18.07%)) or patients with isolated ASD (*p* ≤ 0.001 (34.90% vs. 3.70%)) or patients with epilepsy (*p*=0.004 (34.90% vs. 16.90%)). The proportion of pCNVs in patients with isolated ASD was significantly lower than that in patients with isolated DD/ID (*p* ≤ 0.001 (3.70% vs. 18.07%)) or patients with ASD (*p*=0.007 (3.70% vs. 16.90%)).

### 3.2. Microdeletion/Microduplication Syndromes

Of the 127 cases with abnormal results, 76 cases had 35 types of microdeletion/microduplication syndromes (59.84%), including Williams-Beuren syndrome, Angelman syndrome, Prader–Willi syndrome, 22q11 deletion syndrome (velocardiofacial/DiGeorge syndrome), and Wolf–Hirschhorn syndrome. Twenty-nine microdeletion/microduplication syndromes were detected in patients with isolated DD/ID (8.73%, 29/332), 40 in patients with MCA (26.85%, 40/149), 3 in patients with isolated ASD (3.70%, 3/81), and 5 in patients with epilepsy (7.04%, 5/71) (Table 3).

**Figure 1:**
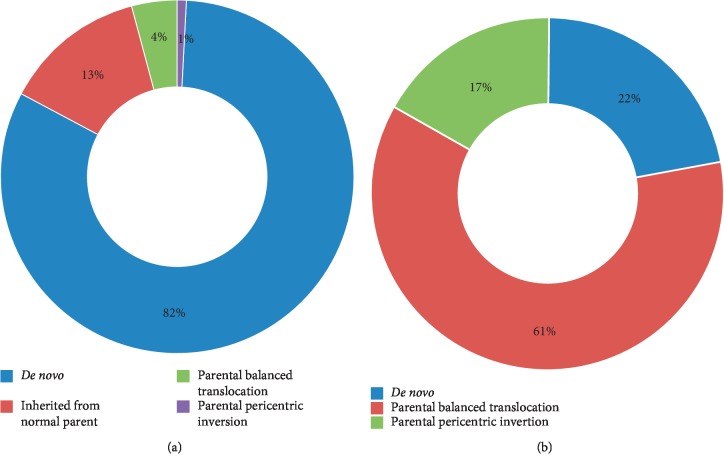
Characterization of pCNVs in the patients with DD/ID. (a) The inheritance of the 77 microdeletion/microduplication syndromes. (b) The inheritance of the 18 submicroscopic unbalance rearrangements.

Most of the microdeletion/microduplication syndromes were *de novo* (63/77), including 2 patients with AS caused by paternal UPD15 and 3 patients with Russell–Silver syndrome (RSS) caused by maternal UPD7. However, some patients inherited neurocognitive disorder susceptibility loci, including 16p11.2 recurrent microdeletion (1/3) and 16p13.11 recurrent microduplication/microdeletion (2/4) from their normal parents. Two male patients had maternally inherited Xq28 (MECP2) duplication, and 1 male patient had maternally inherited Xp11.22-linked intellectual disability. All 3 cases with 15q11-q13 duplication syndrome were inherited from their normal mothers, in which one also suffered from 16p11.2 recurrent microdeletion inherited from her normal father (Figure 1(a)).

### 3.3. Submicroscopic Unbalance Rearrangements

Of the 127 cases with abnormal results, 18 cases were detected with submicroscopic unbalance rearrangements (14.17%), including 10 cases inherited from parental balanced translocations or pericentric inversions (Figure 1(b)). Fifteen cases had subtelomeric aberrations at the end of two different chromosomes, of which 8 cases were inherited from normal parents with balanced translocations confirmed by karyotyping. Three cases had subtelomeric aberrations at both ends of the same chromosome, of which 2 cases were inherited from normal parents with pericentric inversions confirmed by karyotyping.

## 4. Discussion

The establishment of genetic etiological diagnoses for DD/ID children is usually challenging due to the high frequency of relatively nonspecific symptoms shared by numerous potential syndromes. We identified pCNVs in 20.06% of cases, which was comparable to other reported series [8, 14–17]. Interestingly, our study revealed some new findings with certain clinical significance.

### 4.1. More Deletions than Duplications in pCNVs

In our study, the proportion of deletions was extremely higher than duplications in pCNVs. This finding is consistent with the notion of Ruderfer et al. [18] that many duplications present in the human genome are benign, and most phenotypically normal individuals possess a higher number of duplications than deletions. The dosage-sensitive genes have the ability to cause phenotypes [9]. In our study, 32 genes were confirmed with “sufficient evidence for haploinsufficiency” in the pathogenic deletions, while only 2 genes were confirmed with “sufficient evidence for triplosensitivity” in the pathogenic duplications (https://www.clinicalgenome.org/), which influenced the phenotypes of these patients. Thus, deletions contributed more pathogenic interpretations than duplications.

### 4.2. Diagnostic Yields Associated with the Phenotypes

The diagnostic yield of pCNVs (including microdeletion/microduplication syndromes) in the MCA subgroup was significantly higher than that in the other 3 subgroups, which implied that severe and complex phenotypes, such as dysmorphology or congenital anomalies, tend to have a higher likelihood of identifying a genetic etiology [4]. Case 92 is a 13-year-old female who has mild ID, specifically a learning disability with a cleft palate. CMA revealed a 5242-kb duplication in the 15q11.2q13.1 (15q11-q13 duplication syndrome) inherited from her normal mother and a 748-kb deletion in 16p11.2 (16p11.2 recurrent microdeletion) inherited from her normal father. Evidence suggests that maternally derived 15q11.2q13.1 duplications are more frequently associated with abnormal phenotypes [19]. Weiss et al. [20] reported that the phenotype of 16p11.2 recurrent microdeletion is characterized by DD, ID, and/or ASD. It is rare that one patient suffers from two different microdeletion/microduplication syndromes. We hypothesized that both the duplication and deletion contributed to the phenotype of the patient. The probability of her parents having another baby with one of the pCNVs or for both is extremely as high as 75%. Wolfe et al. [21] identified that 16p11.2 deletions and 15q11.2q13.1 duplications had incomplete penetrance with high frequencies in neurodevelopmental disorders; however, they sometimes can be observed in healthy controls. So, the phenotype of the baby with pCNV(s) could not be confirmed before birth.

In the isolated DD/ID subgroup and DD/ID with epilepsy subgroup, the diagnostic yields of pCNVs were significantly lower than those of the MCA subgroup but significantly higher than those of the isolated ASD subgroup. The more phenotypes the patients had, such as epilepsy, the higher the likelihood of finding a genetic etiology [9]. However, the diagnostic yields of pCNVs between these two subgroups were not statistically significant. Next-generation sequencing (NGS) also contributes to the identification of epilepsy caused by monogenic mutations [22], which might be omitted by CMA.

The diagnostic yield of pCNVs was significantly lower in the patients with isolated ASD than in the other 3 subgroups, which was consistent with the results of Ho et al. [16]. We assumed that some other genetic etiologies, such as single-gene disorders, may contribute to the pathogenesis of ASD, which requires further investigation. We detected 3 microdeletion/microduplication syndromes in this subgroup, including Smith–Magenis syndrome, Potocki–Lupski syndrome, and 2q37 monosomy, which were reported in the previous studies [23, 24].

Thus, we believe that the correct genetic diagnosis confirmed by CMA is imperative to medical management and prognostic evaluation of patients with DD/ID.

### 4.3. Assessment of Recurrence Risks

In our study, microdeletion/microduplication syndromes were detected in 76 patients. As most of the syndromes are *de novo* (63/77), the recurrence risk of these sporadic syndromes is extremely low. However, the parents of the DD/ID patients with maternally derived 15q11-q13 duplication (Cases 23, 24, and 92) or some parentally derived recurrent CNVs such as 16p11.2 microdeletion (Case 92) or 16p13.11 microduplication/microdeletion (Cases 22 and 86) have a recurrence risk of 50%. In addition, the parents of male patients with maternally derived X-chromosomal aberrations including Xp22.31 deletion, Xq28 duplication, or Xp11.22 duplication have a recurrence risk of 25%. Hence, the CMA results of these parents are more vital to evaluate the recurrence risk in reproduction.

In the 127 cases with pCNVs, 18 cases (14.17%) were identified with submicroscopic subtelomeric aberrations, including 7 patients suffering from microdeletion/microduplication syndromes, which was consistent with the results of Cheng et al. [25]. In the 18 cases, 8 families were confirmed with parental balanced translocations and 2 families were confirmed with pericentric inversions by karyotyping. These families have an extremely high risk of having another child with submicroscopic subtelomeric aberrations induced DD/ID (10/18). Conventional cytogenetics can only recognize chromosomal rearrangements with a limited resolution of 5∼10 Mb [9]. There were still 8 cases diagnosed as *de novo* submicroscopic subtelomeric aberrations by comparing with the karyotypes of their parents. These parents should be further tested whether they have balanced translocations or pericentric inversions by locus specific FISH probes according to the results of CMA. Fortunately, all the 18 families may possibly have a healthy child if effective genetic counseling was given based on reasonable techniques of prenatal or preimplantational diagnosis.

### 4.4. Limitations of CMA

Parental study is usually indispensable because it not only helps with the interpretation of the clinical significance of CNVs but also contributes to genetic counseling and the evaluation of recurrence risk of genetic abnormalities [26]. However, even though the results of normal parents were compared with their children, there was still 1.11% VUS in our study. In general, the rate of VUS will decrease as more CMA results are obtained from the normal parents. The establishment of a normal individual CMA database might be helpful to address this issue.

CMA has been confirmed as a vital technology to offer extremely higher diagnostic yield compared with chromosomal karyotype analysis in DD/ID. However, the genetic etiology of approximately 80% of patients remains unknown. Development of NGS offers another option for the genetic diagnosis of DD/ID. Currently, with an increased number of pathogenic mutations of genes associated with DD/ID detected by NGS, the diagnostic yield could be further improved by 20∼30% [27, 28]. A combination of CMA and NGS could be a comprehensive strategy, but the cost-effectiveness should be considered.

## Figures and Tables

**Table 1:** Summary of CMA results in 633 patients.

Category	Microarray results (%)			Total
	Pathogenic CNVs	VUS	Normal	
Isolated DD/ID	60 (18.07)	5 (1.51)	267 (80.42)	332
DD/ID with MCA	52 (34.90)	0 (0.00)	97 (65.10)	149
DD/ID with ASD	3 (3.70)	0 (0.00)	78 (96.30)	81
DD/ID with epilepsy	12 (16.90)	2 (2.82)	57 (80.28)	71
Total	127 (20.06)	7 (1.11)	499 (78.83)	633

DD: developmental delay; ID: intellectual disability; MCA: multiple congenital anomaly; ASD: autism spectrum disorder.

**Table 2:** Characteristics of pCNVs detected by CMA among the 127 patients.

No.	Clinical feature	Age	Gender	CMA results	Sizes of CNVs (kb)	Copy number	Syndromes	OMIM gene	Inherited or *de novo*
1	ID	17 y	F	arr[GRCh37] 12p12.1(21369190_25634175)x1	3995	Loss	Lamb-Shaffer syndrome	SOX5	*de novo*
2	DD	3 y	F	arr[GRCh37] 4p16.3p16.1(68345_8066350)x1	7998	Loss	Wolf–Hirschhorn syndrome		*de novo*
3	ID	5 y	M	arr[GRCh37] 7q11.23(72723370_74136633)x1	1413	Loss	Williams-Beuren syndrome	ELN	*de novo*
4	DD	4 y	M	arr[GRCh37] Xq28(153118233_153878720)x2	760	Gain	Xq28 (MECP2) duplication	MECP2	Inherited from normal mother
5	ID	5 y	M	arr[GRCh37] 15q11.2q26.3(22817870_102397317)hmz	79,579	LOH (paternal UPD15)	Angelman syndrome	UBE3A	*de novo*
6	DD	4 y	M	arr[GRCh37] 7q11.23(72718123_74136633)x1	1419	Loss	Williams-Beuren syndrome	ELN	*de novo*
7	DD	19 m	M	arr[GRCh37] 15q11.2q13.1(23632677_28704050)x1	5071	Loss	Angelman syndrome	UBE3A	*de novo*
8	ID	16 y	F	arr[GRCh37] 7q11.23(72718123_74141494)x1	1423	Loss	Williams-Beuren syndrome	ELN	*de novo*
9	DD	16 m	M	arr[GRCh37] 11p11.2(44506359_47897669)x1	3391	Loss	Potocki–Shaffer syndrome	MYBPC3	*de novo*
10	ID	6 y	M	arr[GRCh37] 15q11.2q13.1(23290787_28526905)x1	5147	Loss	Angelman syndrome	UBE3A	*de novo*
11	ID	7 y	M	arr[GRCh37] Xq28(153030708_155233098)x2	2202	Gain	Xq28 (MECP2) duplication	MECP2	Inherited from normal mother
12	ID	16 y	F	arr[GRCh37] 15q11.2q26.3(22817870_102397317)hmz	79,579	LOH (paternal UPD15)	Angelman syndrome	UBE3A	*de novo*
13	ID	6 y	M	arr[GRCh37] 7q11.23 (72611954_75147402)x1	1745	Loss	Williams-Beuren syndrome	ELN	*de novo*
14	ID	5 y	M	arr[GRCh37] 16p13.3(85880_2045435)x1	1960	Loss	ATR-16 syndrome		*de novo*
15	DD	17 m	F	arr[GRCh37] 7q11.23(72692112_74184702)x1	1496	Loss	Williams-Beuren syndrome	ELN	*de novo*
16	ID	16 y	F	arr[GRCh37] 22q13.33(50974299_51197766)x1	223	Loss	22q13 deletion syndrome (Phelan–Mcdermid syndrome)	SHANK3	*de novo*
17	ID	9 y	F	arr[GRCh37] 17p11.2 (16761814_20304118)x3	3542	Gain	Potocki–Lupski syndrome (17p11.2 duplication syndrome)		*de novo*
18	DD	9 m	F	arr[GRCh37] 7q11.23(72723370_74136633)x1	1413	Loss	Williams-Beuren syndrome	ELN	*de novo*
19	ID	6 y	F	arr[GRCh37] 22q13.31q13.33(48234841_51197766)x1	2963	Loss	22q13 deletion syndrome (Phelan–Mcdermid syndrome)	SHANK3	*de novo*
20	DD	13 m	F	arr[GRCh37] 22q11.21(18919477_21436003)x3	2516	Gain	22q11 duplication syndrome		*de novo*
21	ID	9 y	M	arr[GRCh37] 7q11.23(72723370_74136633)x1	1413	Loss	Williams-Beuren syndrome	ELN	*de novo*
22	ID	12 y	M	arr[GRCh37] 16p13.11(14892975_16538596)x3	1646	Gain	16p13.11 recurrent microduplication (neurocognitive disorder susceptibility locus)		Inherited from normal mother
23	ID	5 y	F	arr[GRCh37] 15q11.2q13.1(22770421_28560664)x3	5790	Gain	15q11-q13 duplication syndrome		Inherited from normal mother
24	ID	17 y	F	arr[GRCh37] 15q11.2q13.1(22770421_28526905)x3	5756	Gain	15q11-q13 duplication syndrome		Inherited from normal mother
25	DD	13 m	F	arr[GRCh37] 5q23.3q31.2(129203365_139475046)x3	10,272	Gain			*de novo*
26	ID	16 y	F	arr[GRCh37] 8p23.3p23.1(158048_9781509)x1	9623	Loss	8p23.1 deletion syndrome	CSMD1	de novo
27	ID	9 y	F	arr[GRCh37] 7q36.1q36.3(151376795_159119707)x1	7743	Loss		SHH; KMT2C; DPP6; MNX1	*de novo*
28	DD	3 y	M	arr[GRCh37] 1q43q44(239750391_249224684)x1	9474	Loss	1q43-q44 deletion syndrome	CHRM3; AKT3; HNRNPU	*de novo*
29	ID	12 y	M	arr[GRCh37] 3q23q25.1(141486765_151354816)x1	9868	Loss		ZIC1; ZIC4	*de novo*
30	DD	4 y	F	arr[GRCh37] 18p11.32p11.21(136227_12342194)x1	12,206	Loss		TGIF1	*de novo*
31	ID	16 y	F	arr[GRCh37] 11q24.2q25(124419306_134937416)x1	10,518	Loss			*de novo*
32	ID	17 y	F	arr[GRCh37] 3q27.3q29(187068732_194767726)x1	7699	Loss		TP63; FGF12	*de novo*
33	DD	8 m	M	arr[GRCh37] 10q26.13q26.3(123584147_135426386)x1	11,842	Loss		EBF3	*de novo*
34	DD	3 y	M	arr[GRCh37] 11q14.1(77492774_85312824)x1	7820	Loss		DLG2	*de novo*
35	DD	4 y	M	arr[GRCh37]Mosaic 15q14q24.1(35050247_75972909)x1.63	40,923	Loss (Mosaic)	15q24 recurrent microdeletion syndrome		*de novo*
36	DD	4 y	M	arr[GRCh37] 1q42.13q44(228801122_249181598)x3	20,380	Gain			*de novo*
37	ID	6 y	M	arr[GRCh37] 1q42.13q44(229917977_249224684)x3	19,307	Gain			*de novo*
38	DD	3 y	F	arr[GRCh37] 12p13.33q12(173786_40931729)x3	40,758	Gain	Partial chromosome 12 trisomy		*de novo*
39	ID	8 y	M	arr[GRCh37] Xp21.3p11.23(27954516_48270449)x1	20,316	Loss	Xp11.23 region (includes MAOA and MAOB)		*de novo*
40	ID	10 y	F	arr[GRCh37] 18q21.32q23(58617060_78013728)x1	19,847	Loss			*de novo*
41	ID	16 y	F	arr[GRCh37] 11q14.2q22.3(87455736_109777755)x1	22,322	Loss			*de novo*
42	DD	4 y	F	arr[GRCh37] 4p16.3p15.31(290685_18118492)x3	17,828	Gain	4p16.3 terminal (Wolf–Hirschhorn syndrome) region		*de novo*
				arr[GRCh37] 4q34.1q35.2(176152080_190957460)x1	14,805	Loss			
43	DD	3 y	M	arr[GRCh37] 8p23.3p23.1(158048_10915395)x3	10,757	Gain	8p23.1 duplication syndrome	SOX7	*de novo*
				arr[GRCh37] 9p24.3p24.1(208454_6308953)x1	6100	Loss		DMRT1	
44	ID	8 y	M	arr[GRCh37] 4p16.3p16.1(68345_9514461)x3	9446	Gain	4p16.3 terminal (Wolf–Hirschhorn syndrome) region		Paternal balanced translocation 46,XY,t(4; 8) (p16q23)
				arr[GRCh37] 8p23.3p23.1(158048_7044046)x1	6886	Loss		CSMD1	
45	ID	17 y	F	arr[GRCh37] 9p24.3p24.1(208454_8748943)x3	8540	Gain			*de novo*
				arr[GRCh37] 18q22.1q23(65906752_78013728)x1	12,107	Loss			
46	ID	3 y	M	arr[GRCh37] 6q27(169727875_170914297)x3	1186	Gain			*de novo*
				arr[GRCh37] 13q33.3q34(107636085_115107733)x1	7472	Loss		CHAMP1; BSVD2	
47	ID	17 y	M	arr[GRCh37] 11q25(131001110_134937416)x1	3936	Loss			Maternal balanced translocation 46,XX,t(11; 18) (q25; q21.2)
				arr[GRCh37] 18q21.2q23(50912872_78013728)x3	27,101	Gain			
48	ID	16 y	F	arr[GRCh37] 9p24.3p21.1(208454_30555044)x3	30,347	Gain			Paternal balanced translocation 46,XY,t(9; 18) (p21; p11.3)
				arr[GRCh37] 18p11.32p11.31(136227_5485196)x1	5349	Loss		TGIF1	
49	ID	7 y	M	arr[GRCh37] 3p26.3p26.1(61891_5189701)x1	5128	Loss		CNTN4; CNTN6; ITPR1	*de novo*
				arr[GRCh37] 7q33q36.3(134287922_159119707)x3	24,832	Gain		SHH	
50	DD	3 y	F	arr[GRCh37] 6q25.3q27(159131590_170914297)x3	11,783	Gain			Maternal balanced translocation 46,XX,t(6; 10) (q25.3; p15.3)
				arr[GRCh37] 10p15.3(100047_1947393)x1	1847	Loss		ZMYND11	
51	ID	16 y	F	arr[GRCh37] 9p24.3p13.3(208454_33702198)x3	33,494	Gain			*de novo*
				arr[GRCh37] 19p13.3(260911_1247822)x1	987	Loss			
52	DD	3 y	M	arr[GRCh37] 12q12(44719567_46210900)x1	1491	Loss		ARID2	*de novo*
53	ID	7 y	F	arr[GRCh37] Xq27.3q28(145269560_149282242)x1	4013	Loss		FMR1; AFF2; IDS	*de novo*
54	DD	4 y	M	arr[GRCh37] 2q22.3(144457537_145255844)x1	798	Loss		ZEB2	*de novo*
55	ID	16 y	M	arr[GRCh37] Xq28(154476199_155233098)x1	759	Loss		RAB39B	Inherited from normal mother
56	ID	10 y	M	arr[GRCh37] 8p11.22(38344498_39172014)x3	8575	Gain			*de novo*
57	ID	10 y	M	arr[GRCh37] 1p36.33p36.32(1156338_2468052)x1	1302	Loss		GNB1; GABRD	*de novo*
58	ID	14 y	F	arr[GRCh37] 9q34.11(131231815_132005416)x1	774	Loss		SPTAN1	*de novo*
59	ID	17 y	F	arr[GRCh37] 6q27(169471201_170914297)x1	1443	Loss		ERMARD; TBP	*de novo*
60	ID	12 y	F	arr[GRCh37] 1p36.33p36.32(849466_2516031)x1	1667	Loss		GNB1; GABRD	*de novo*
61	DD + MCA (short status)	3 y	M	arr[GRCh37] Xp11.22(53359258_53647606)x2	288	Gain	Xp11.22-linked intellectual disability	HUWE1	Inherited from normal mother
62	DD + MCA (microtia, cleft palate, ventricular septal defect)	8 m	F	arr[GRCh37] 4p16.3(68345_3488721)x1	3420	Loss	Wolf–Hirschhorn syndrome		*de novo*
63	DD + MCA (facial dysmorphism, supravalvular aortic stenosis (SVAS) and supravalvular pulmonary stenosis)	11 m	M	arr[GRCh37] 7q11.23(72718123_74136633)x1	1419	Loss	Williams-Beuren syndrome	ELN	*de novo*
64	ID + MCA (facial dysmorphism, short status)	6 y	M	arr[GRCh37] 17p11.2(16657318_20287758)x1	3630	Loss	Smith–Magenis syndrome	RAI1; FLCN	*de novo*
65	DD + MCA (facial dysmorphism, short status)	9 m	M	arr[GRCh37] 7q11.23(72697461_74136633)x1	1439	Loss	Williams-Beuren syndrome	ELN	*de novo*
66	ID + MCA (facial dysmorphism, short status)	16 y	F	arr[GRCh37] 17p11.2(16736261_20417235)x1	3681	Loss	Smith–Magenis syndrome	RAI1; FLCN	*de novo*
67	ID + MCA (facial dysmorphism, cleft palate, short status)	6 y	F	arr[GRCh37] 7q11.23(72713282_74154209)x1	1441	Loss	Williams-Beuren syndrome	ELN	*de novo*
68	DD + MCA (facial dysmorphism, muscular hypotonia)	2 y	F	arr[GRCh37] 7p22.3p11.1(50943_58019983)hmz	57,969	LOH (maternal UPD7)	Silver–Russell syndrome		*de novo*
69	ID + MCA (ventricular septal defect)	5 y	F	arr[GRCh37] 15q11.2q13.1(22770421_28704050)x1	5934	Loss	Prader–Willi syndrome	UBE3A	*de novo*
70	DD + MCA (facial dysmorphism, short status)	16 m	M	arr[GRCh37] 7q11.23(72697239_74136633)x1	1439	Loss	Williams-Beuren syndrome	ELN	*de novo*
71	DD + MCA (facial dysmorphism, hypoplasia of the corpus callosum, ventricular septal defect, short status)	9 m	M	arr[GRCh37] 17p13.3(525_2780094)x1	2780	Loss	Miller–Dieker syndrome	PAFAH1B1	de novo
72	DD + MCA (facial dysmorphism, supravalvular aortic stenosis (SVAS), ventricular septal defect)	9 m	M	arr[GRCh37] 7q11.23(72713282_74136633)x1	1423	Loss	Williams-Beuren syndrome	ELN	*de novo*
73	DD + MCA (muscular hypotonia, dysphagia, cryptorchidism)	3 m	M	arr[GRCh37] 15q11.2q13.1(23290787_28540345)x1	5250	Loss	Prader–Willi syndrome	UBE3A	*de novo*
74	DD + MCA (triangular shaped face, short status, body asymmetry)	13 m	F	arr[GRCh37] 7p22.3p11.1(50943_58019983)hmz	57,969	LOH (maternal UPD7)	Silver–Russell syndrome		*de novo*
75	DD + MCA (facial dysmorphism, cafe-au-lait spots, atrial septal defect)	18 m	M	arr[GRCh37] 17q11.2(29025996_30369402)x1	1343	Loss	NF1-microdeletion syndrome	NF1	*de novo*
76	ID + MCA (facial dysmorphism, short status)	13 y	F	arr[GRCh37] 5p15.33p15.31(113576_9756329)x1	9643	Loss	Cri du chat syndrome (5p deletion)		*de novo*
77	ID + MCA (facial dysmorphism, brachydactyly)	9 y	F	arr[GRCh37] 2q37.3(239755969_242782258)x1	3026	Loss	2q37 monosomy	HDAC4	*de novo*
78	DD + MCA (hypertelorism, overgrowth)	5 m	F	arr[GRCh37] 15q24.3q26.3(78160033_102429040)x3	24,269	Gain	15q26 overgrowth syndrome		Paternal balanced translocation 46,XY,t(3; 15) (p26; q24)
				arr[GRCh37] 3p26.3(61891_1542088)x1	1480	Loss		CNTN6	
79	DD + MCA (facial dysmorphism, esophageal atresia, external auditory canal atresia)	7 m	M	arr[GRCh37] 22q13.31q13.33(48283717_51197766)x1	2914	Loss	22q13 deletion syndrome (Phelan–Mcdermid syndrome)	SHANK3	*de novo*
				arr[GRCh37] 9q34.2q34.3(136244652_141018648)x3	4774	Gain		EHMT1	
80	ID + MCA (atrial septal defect, cleft palate, hearing impairment)	5 y	M	arr[GRCh37] 22q11.1q11.21(16888899_20716903)x3	3828	Gain	Cat eye syndrome		Maternal balanced translocation 46,XX,t(11; 22) (q23.3; q11.2)
				arr[GRCh37] 11q23.3q25(116683754_134937416)x3	18,254	Gain			
81	DD + MCA (polysyndactyly)	7 m	M	arr[GRCh37] 16p11.2(29351825_30176508)x1	825	Loss	16p11.2 recurrent microdeletion		*de novo*
82	DD + MCA (triangular shaped face, short status, muscular hypotonia)	14 m	F	arr[GRCh37] 7p22.3p11.1(50943_58019983)hmz	57,969	LOH (maternal UPD7)	Silver–Russell syndrome		*de novo*
83	ID + MCA (atrial septal defect, ventricular septal defect)	9 y	M	arr[GRCh37] 22q11.21(18648855_21800471)x1	3152	Loss	22q11 deletion syndrome (velocardiofacial/DiGeorge syndrome)	TBX1	*de novo*
84	DD + MCA (short status)	3 y	M	arr[GRCh37] 15q11.2q13.1(23290787_28928730)x1	5638	Loss	Angelman syndrome	UBE3A	*de novo*
85	ID + MCA (congenital heart disease, polysyndactyly)	16 y	F	arr[GRCh37] 22q11.21(18648855_21800471)x1	3152	Loss	22q11 deletion syndrome (velocardiofacial/DiGeorge syndrome)	TBX1	*de novo*
86	DD + MCA (facial dysmorphism)	13 m	M	arr[GRCh37] 16p13.11(14913788_16282869)x3	1369	Gain	16p13.11 recurrent microduplication (neurocognitive disorder susceptibility locus)		Inherited from normal mother
87	DD + MCA (muscular hypotonia, ventricular septal defect, cryptorchidism)	3 m	M	arr[GRCh37] 15q11.2q13.1(23290787_28540345)x1	5250	Loss	Prader–Willi syndrome	UBE3A	*de novo*
88	DD + MCA (cleft palate)	3 y	M	arr[GRCh37] 16p11.2(29428531_30176508)x1	748	Loss	16p11.2 recurrent microdeletion		*de novo*
89	ID + MCA (facial dysmorphism, cleft palate, polysyndactyly, short status)	11 y	M	arr[GRCh37] 17q21.31q21.32(43170339_44988790)x1	1818	Loss	17q21.31 recurrent microdeletion syndrome (Koolen–de Vries syndrome)	KANSL1	*de novo*
90	ID + MCA (short status)	9 y	F	arr[GRCh37] 22q11.21(18648855_21800471)x1	3169	Loss	22q11 deletion syndrome (velocardiofacial/DiGeorge syndrome)	TBX1	*de novo*
91	DD + MCA (cleft palate)	3 y	F	arr[GRCh37] 16p13.11(15481747_16390970)x3	909	Gain	16p13.11 recurrent microduplication (neurocognitive disorder susceptibility locus)		*de novo*
92	ID + MCA (cleft palate)	13 y	F	arr[GRCh37] 15q11.2q13.1(23281885_28526905)x3	5245	Gain	15q11-q13 duplication syndrome		Inherited from normal mother
				arr[GRCh37] 16p11.2(29428531_30176508)x1	748	Loss	16p11.2 recurrent microdeletion		Inherited from normal father
93	DD + MCA (facial dysmorphism, catlike cry, ventricular septal defect, short status)	3 m	F	arr[GRCh37] 5p15.33p13.3(113576_32114177)x1	32,001	Loss	Cri du chat syndrome (5p deletion)	TRIO; CTNND2	*de novo*
94	DD + MCA (short status)	11 m	F	arr[GRCh37] Xp22.33p22.31(168551_8030262)x1	7862	Loss	Leri–Weill dyschondrosteosis (LWD): SHOX deletion	SHOX; ARSE	*de novo*
95	ID + MCA (cleft palate)	6 y	F	arr[GRCh37] 7q11.23(72692112_74154209)x1	1462	Loss	Williams-Beuren syndrome	ELN	*de novo*
96	ID + MCA(micrognathia)	16 y	F	arr[GRCh37] 8p23.3p23.1(158048_10029980)x1	9872	Loss	8p23.1 deletion syndrome	CSMD1	*de novo*
97	ID + MCA (atrial septal defect, microtia, polysyndactyly)	7 y	M	arr[GRCh37] 5q34q35.3(162638031_180329359)x3	17,691	Gain	5q35 recurrent (Sotos syndrome) region (includes NSD1)	FBXW11	Maternal balanced translocation 46,XX,t(5; 12) (q34; p13.32)
				arr[GRCh37] 12p13.33p13.32(173786_4264694)x1	4091	Loss	12p13.33 microdeletion syndrome		
98	DD + MCA (cryptorchidism, short status)	19 m	M	arr[GRCh37] 4q34.1q35.2(174352834_190957460)x3	16605	Gain			*de novo*
				arr[GRCh37] Xp22.33p22.31(168551_6455151)x0	6287	Loss	Leri–Weill dyschondrosteosis (LWD): SHOX deletion	SHOX; ARSE	
99	DD + MCA (gallbladder agenesis)	3 y	F	arr[GRCh37] 8p23.3p23.1(158048_7044046)x1	6686	Loss		CSMD1	*de novo*
				arr[GRCh37] 8p23.1p12(11936000_33616243)x3	21,860	Gain			
100	ID + MCA (atrial septal defect, hypermyotonia)	6 y	M	arr[GRCh37] 2p23.1p22.1(32046639_38823958)x1	6777	Loss		SPAST	*de novo*
101	DD + MCA (hypoplasia of the corpus callosum)	3 m	F	arr[GRCh37] 13q33.2q34(106348324_115107733)x1	8759	Loss		CHAMP1; BSVD2	*de novo*
102	ID + MCA (micrognathia, polysyndactyly)	14 y	F	arr[GRCh37] 18p11.32q11.2(136227_20989843)x3	20,854	Gain			Maternal balanced translocation 46,XX,t(18; 21) (q11.2; q21)
				arr[GRCh37] 21q11.2q21.1(15016486_20371429)x1	5355	Loss			
103	DD + MCA (hypermyotonia, blepharophimosis)	3 m	M	arr[GRCh37] 3q22.1q23(132876177_139772196)x1	6896	Loss		FOXL2	*de novo*
104	DD + MCA (facial dysmorphism)	8 m	F	arr[GRCh37] 10p15.3p12.2(100047_23162330)x3	23,062	Gain			Paternal inversion 46,XY,inv(10) (p12q26)
				arr[GRCh37] 10q26.3(134248768_135426386)x1	1178	Loss			
105	DD + MCA (ventricular septal defect, aortic stenosis)	21 m	M	arr[GRCh37] 7p21.1p11.2(16641066_56373573)x3	39,733	Gain			*de novo*
				arr[GRCh37] 4q13.1q13.2(65818383_68116457)x1	2298	Loss			
106	DD + MCA (facial dysmorphism, cryptorchidism)	3 y	M	arr[GRCh37] 3q13.33q25.1(121200603_151876470)x3	30,676	Gain			*de novo*
107	ID + MCA (facial dysmorphism)	3 y	M	arr[GRCh37] 14q12(28897081_31268243)x1	2371	Loss	Rett syndrome	FOXG1	*de novo*
108	DD + MCA (atrial septal defect)	14 m	F	arr[GRCh37] 20p13(61661_2150330)x1	2089	Loss		CSNK2A1; PDYN	*de novo*
109	DD + MCA (facial dysmorphism, overgrowth, body asymmetry)	2 y	F	arr[GRCh37] Xq21.31q27.3(86577241_145860589)x3	59,283	Gain	Pelizaeus–Merzbacher disease (carrier)	PLP1	*de novo*
110	DD + MCA (cryptorchidism, hypospadias)	3 m	M	arr[GRCh37] 5p14.3p12(19454082_45506818)x3	26,053	Gain			*de novo*
111	DD + MCA (facial dysmorphism, bilateral single transverse palmar creases)	3 m	F	arr[GRCh37] 9p24.3q13(208454_68216577)x3	10,188	Gain	Chromosome 9p trisomy		*de novo*
112	ID + MCA (facial dysmorphism, webbed neck, low-set ears)	5 y	F	arr[GRCh37] Xp22.33p11.22(168551_52706689)x1	52,538	Loss			*de novo*
				arr[GRCh37] Xp11.22q28(52833230_155233098)x3	102,400	Gain			
113	ID + ASD	13 y	F	arr[GRCh37] 17p11.2(16657318_20463423)x1	3806	Loss	Smith–Magenis syndrome	RAI1; FLCN	*de novo*
114	DD + ASD	3 y	M	arr[GRCh37] 17p11.2 (16745600_20417235)x3	3672	Gain	Potocki–Lupski syndrome (17p11.2 duplication syndrome)		*de novo*
115	ID + ASD	10 y	M	arr[GRCh37] 2q37.2q37.3(235790877_242782258)x1	6991	Loss	2q37 monosomy	HDAC4	Paternal inversion 46,XY,inv(2) (p24q37.2)
				arr[GRCh37] 2p25.3p24.3(12770_12658812)x3	12,646	Gain			
116	ID + epilepsy (ichthyosis)	12 y	M	arr[GRCh37] Xp22.31(6455151_8141076)x0	1686	Loss	Steroid sulphatase deficiency (STS)	STS	Inherited from normal mother
117	ID + epilepsy	8 y	M	arr[GRCh37] 15q11.2q13.1(22770421_28704050)x1	5934	Loss	Angelman syndrome	UBE3A	*de novo*
118	DD + epilepsy	3 y	F	arr[GRCh37] 16p13.12p13.11(14777379_16533107)x1	1756	Loss	16p13.11 recurrent microdeletion (neurocognitive disorder susceptibility locus)		*de novo*
119	ID + epilepsy	6 y	M	arr[GRCh37] 15q11.2q13.1(23620191_28526905)x1	4907	Loss	Angelman syndrome	UBE3A	*de novo*
120	DD + epilepsy	3 y	F	arr[GRCh37] 16p11.2(28557432_30176508)x1	1619	Loss	16p11.2 microduplication syndrome	SH2B1	*de novo*
121	DD + epilepsy	11 m	F	arr[GRCh37] 20q13.33(61485437_62790113)x1	1305	Loss		CHRNA4; KCNQ2	*de novo*
122	ID + epilepsy	5 y	F	arr[GRCh37] 13q33.3q34(108237906_115107733)x1	6870	Loss		CHAMP1; BSVD2	*de novo*
123	DD + epilepsy	4 y	F	arr[GRCh37] Xq23q24(111170674_117964845)x1	6794	Loss			*de novo*
124	ID + epilepsy	8 y	M	arr[GRCh37] Xp22.13p21.3(17125886_28993521)x2	11,868	Gain			*de novo*
125	ID + epilepsy	6 y	M	arr[GRCh37] 2p24.3p24.2(15850097_16790467)x1	940	Loss		MYCN	*de novo*
126	ID + epilepsy	3 y	M	arr[GRCh37] 2q24.3(164444391_168745074)x1	4301	Loss		SCN1A; SCN2A; SCN9A	*de novo*
127	ID + epilepsy	6 y	M	arr[GRCh37] 1p36.33(849466_2226509)x1	1377	Loss		GNB1; GABRD	*de novo*

LOH: loss of heterozygosity; UPD: uniparental disomy.

**Table 3:** Microdeletion/microduplication syndromes in the 76 patients.

Syndromes	Isolated DD/ID	DD/ID with MCA	DD/ID with ASD	DD/ID with epilepsy	Total
Williams-Beuren syndrome	7	6	0	0	13
Angelman syndrome	4	1	0	2	7
Silver–Russell syndrome	0	3	0	0	3
15q11-q13 duplication syndrome	2	1	0	0	3
16p11.2 recurrent microdeletion	0	3	0	0	3
16p13.11 recurrent microduplication (neurocognitive disorder susceptibility locus)	1	2	0	0	3
22q11 deletion syndrome (velocardiofacial/DiGeorge syndrome)	0	3	0	0	3
8p23.1 deletion syndrome	2	1	0	0	3
Prader–Willi syndrome	0	3	0	0	3
Smith–Magenis syndrome	0	2	1	0	3
22q13 deletion syndrome (Phelan–Mcdermid syndrome)	2	1	0	0	3
2q37 monosomy	0	1	1	0	2
Cri du chat syndrome (5p deletion)	0	2	0	0	2
Leri–Weill dyschondrosteosis (LWD): SHOX deletion	0	2	0	0	2
Potocki–Lupski syndrome (17p11.2 duplication syndrome)	1	0	1	0	2
Wolf–Hirschhorn syndrome	1	1	0	0	2
Xq28 (MECP2) duplication	2	0	0	0	2
Cat eye syndrome	0	1	0	0	1
12p13.33 microdeletion syndrome	0	1	0	0	1
15q24 recurrent microdeletion syndrome	1	0	0	0	1
15q26 overgrowth syndrome	0	1	0	0	1
16p11.2 microduplication syndrome	0	0	0	1	1
16p13.11 recurrent microdeletion (neurocognitive disorder susceptibility locus)	0	0	0	1	1
17q21.31 recurrent microdeletion syndrome (Koolen–de Vries syndrome)	0	1	0	0	1
1q43-q44 deletion syndrome	1	0	0	0	1
22q11 duplication syndrome	1	0	0	0	1
ATR-16 syndrome	1	0	0	0	1
Lamb-Shaffer syndrome	1	0	0	0	1
Miller–Dieker syndrome	0	1	0	0	1
NF1-microdeletion syndrome	1	0	0	0	1
Pelizaeus–Merzbacher disease (carrier)	0	1	0	0	1
Potocki–Shaffer syndrome	1	0	0	0	1
Rett syndrome	0	1	0	0	1
Steroid sulphatase deficiency (STS)	0	0	0	1	1
Xp11.22-linked intellectual disability	0	1	0	0	1
Total	29	40	3	5	77

## Data Availability

The CMA data used to support the findings of this study may be released upon application to Prenatal Diagnosis Center, West China Second University Hospital, Sichuan University, who can be contacted at e-mail of the director of Prenatal Diagnosis Center.
